# Lives saved by Global Fund-supported HIV/AIDS, tuberculosis and malaria programs: estimation approach and results between 2003 and end-2007

**DOI:** 10.1186/1471-2334-10-109

**Published:** 2010-04-30

**Authors:** Ryuichi Komatsu, Eline L Korenromp, Daniel Low-Beer, Catherine Watt, Christopher Dye, Richard W Steketee, Bernard L Nahlen, Rob Lyerla, Jesus M Garcia-Calleja, John Cutler, Bernhard Schwartländer

**Affiliations:** 1 The Global Fund to Fight AIDS, Tuberculosis and Malaria, Chemin Blandonnet 8, 1214 Vernier, Geneva, Switzerland; 2 World Health Organization, Stop TB Department, Geneva, Switzerland; 3 Program for Appropriate Technology in Health, MACEPA, Ferney-Voltaire, France; 4 President's Malaria Initiative, USAID, Washington DC, USA; 5 Joint United Nations Program on HIV/AIDS, Policy, Evidence and Partnerships Department, Geneva, Switzerland; 6 World Health Organization, HIV/AIDS Department, Geneva, Switzerland; 7 Health Metric Network Secretariat, Geneva, Switzerland; 8 Joint United Nations Program on HIV/AIDS, China country office, Beijing, China

## Abstract

**Background:**

Since 2003, the Global Fund has supported the scale-up of HIV/AIDS, tuberculosis and malaria control in low- and middle-income countries. This paper presents and discusses a methodology for estimating the lives saved through selected service deliveries reported to the Global Fund.

**Methods:**

Global Fund-supported programs reported, by end-2007, 1.4 million HIV-infected persons on antiretroviral treatment (ARV), 3.3 million new smear-positive tuberculosis cases detected in DOTS (directly observed TB treatment, short course) programs, and 46 million insecticide-treated mosquito nets (ITNs) delivered. We estimated the corresponding lives saved using adaptations of existing epidemiological estimation models.

**Results:**

By end-2007, an estimated 681,000 lives (95% uncertainty range 619,000-774,000) were saved and 1,097,000 (993,000-1,249,000) life-years gained by ARV. DOTS treatment would have saved 1.63 million lives (1.09 - 2.17 million) when compared against no treatment, or 408,000 lives (265,000-551,000) when compared against non-DOTS treatment. ITN distributions in countries with stable endemic *falciparum *malaria were estimated to have achieved protection from malaria for 26 million of child-years at risk cumulatively, resulting in 130,000 (27,000-232,000) under-5 deaths prevented.

**Conclusions:**

These results illustrate the scale of mortality effects that supported programs may have achieved in recent years, despite margins of uncertainty and covering only selected intervention components. Evidence-based evaluation of disease impact of the programs supported by the Global Fund with international and in-country partners must be strengthened using population-level data on intervention coverage and demographic outcomes, information on quality of services, and trends in disease burdens recorded in national health information systems.

## Background

Only in some five years, the Global Fund to Fight AIDS, Tuberculosis and Malaria (Global Fund) has become a major player in international health development. In 2005, the Global Fund provided 21% of international funding for HIV/AIDS programs in low- and middle-income countries, 67% for tuberculosis (TB), and 64% for malaria[1]. By December 2007, it has approved a total of US$ 10.1 billion proposals, in more than 550 grants in 136 countries, of which US$ 4.8 billion had been disbursed to recipients in 134 countries[2]. As control effort of the three diseases is scaled up, there is now considerable interest to show the scale of health benefits from interventions supported.

For evaluation of disease impact, Global Fund recipients collect epidemiological data on relevant changes in mortality and morbidity. Given the nature of the diseases and interventions and data collection mechanisms, it may take several years before impact becomes detectable. For example, reductions in new HIV infections are inferred retrospectively from prevalence trends over preceding years. WHO and UNAIDS regularly publish regional-level HIV incidence and prevalence estimates, using country surveillance and survey data that often require two years to be released [3-7]. Similarly, all-cause under-five mortality, a key impact indicator for malaria in areas of high endemicity, is often measured in population-based surveys with 3-5 year intervals[8]. Malaria-related mortality reductions become apparent only 3-5 years after they start, with the 'birth history' method[9]. The consensus TB estimation model, applied to high-burden countries by WHO with national TB programs[10,11], relies on patient cohort data about treatment outcomes from two preceding years.

In view of these data limitations, it is still early for the impact of Global Fund support to be fully measured. As an *interim *approach, we estimated the scale of lives saved, by summarizing deaths averted from key service delivery results reported by recipient programs. Interventions considered are antiretroviral treatment (ARV), Directly Observed TB Treatment, short-course (DOTS) and insecticide-treated mosquito nets (ITN). Estimates of their efficacies against mortality are available[7,12-17]. This paper presents and discusses the methodology for this approach with estimates of lives saved through service deliveries reported to the Global Fund between 2003 and end-2007.

## Methods

Lives saved and, for ARV, life-years gained, were estimated using mortality estimation models and assumptions used by UNAIDS, WHO Stop TB and Roll Back Malaria. Models were adapted to use as main input service delivery results available from recipient countries (Table 1**and below)**.

**Table 1:** Assumptions used in estimations of lives saved from Global Fund-supported service deliveries.

*Service*	*Mortality reduction based on population coverage*	*Population coverage estimated from service deliveries*	*Other assumptions*
ARV	Survival of people in need of ARV [25,26]: with ARV: 85% by 12 months, 95% over each next year without treatment: 50% by 12 months, 0% by 24 months.} Regional estimates of the average number of life-years gained per patient-year of (Global Fund-supported) ARV, derived by applying the Spectrum model [26] to UNAIDS estimates of national HIV prevalence, HIV mortality and ARV coverage (see Table 2) [24].	All people put on ARV are in need of ARV Those on ARV as of December 2004 started treatment in 2004 (rather than earlier)Of people starting ARV in a given calendar year, starting dates are evenly distributed over that calendar year.	Region-specific 95% URs on mortality estimates from the *Spectrum *model [25,26]

DOTS	Death rates for newly detected smear-positive cases (WHO Stop TB department, unpublished data): DOTS: HIV-positive: country-specific (cross-country average 11%; 95% UR 6-21%) HIV-negative: country-specific (cross-country average 11%; 95% UR 6-20%) Scenario (a) - No TB treatment: HIV-positive: country-specific (cross-country average 83%; 95% UR 70-99%) HIV-negative: country-specific (cross-country average 70%; 95% UR 55-75%) Scenario (b) - non-DOTS treatment: HIV-positive: country-specific (cross-country average 31%; 95% UR 21-43%) HIV-negative: country-specific (cross-country average 24%; 95% UR 15-34%)	All smear-positive cases reported as detected are DOTS-treated Half of lives saved occur in the year of reported case detection, and half in the next year.	For programs not reporting the smear status of new cases, 50% were assumed to be smear-positive Proportions of smear-positive TB cases that are HIV-positive are country-specific estimates, with a cross-country average of 12.3% (95% UR 10-15%) in 2006 [28].

ITNs	All-cause under-5 mortality falls by 5.5 (95% UR 3.4-7.7) per 1000 child-years of protection by ITNs, in areas of stable endemic *falciparum *malaria [29]	Each ITN distributed in a country with stable endemic falciparum malaria protects on average 0.73 child under-5 at risk [31]. Effective lifespan of an ITN is 1.5 years. → Average of 1.1 child-year (0.55-2.2) of protection per ITN distributed, over an ITN's lifetime ITNs reported in a given calendar year count as providing protection for half of that year and (a maximum of) 1.0 year thereafter.	

Abbreviations: ARV = antiretroviral treatment; DOTS = directly observed treatment, short course; ITN = insecticide-treated mosquito net (including long-lasting insecticide nets). Numbers in brackets are 95% uncertainty ranges (UR).

Note: For ARV, in view of its life-extending but non-curative nature, 'lives saved' should be read to mean: the difference between cumulative deaths with and without ARV, i.e. the deaths averted or postponed due to ARV by Global Fund-supported programmes, over the reference period between 2003 and December 2007.

### Service delivery results

Supported programs regularly report progress against targets on standardized indicators[18]http://www.theglobalfund.org, as the basis for performance-based funding[19]. Reported results are verified by independent contractors. The Global Fund secretariat then aggregates grant delivery results across the portfolio, for reporting back to donors[20,21] (Table 2). For ARV, the validity and extent of overlap are assessed with WHO and the US President's Emergency Program for AIDS Relief (PEPFAR), resulting in harmonized numbers[22-24]. Results from multi-country grants were excluded for simplicity.

**Table 2:** Results of three key service deliveries for Global Fund-supported programs

	Dec-04	Dec-05	Dec-06	Dec-07
Current ARVs	130,000	384,000	770,000	1,450,000
Cumulative DOTS	385,000	1,000,000	2,000,000	3,310,000
Cumulative ITNs	1,350,000	7,700,000	18,000,000	45,600,000

ARV = antiretroviral treatment; DOTS = directly observed TB treatment, short course; ITN = insecticide-treated mosquito net (including long-lasting ITNs).

### AIDS mortality averted due to ARV

ARV programs routinely report to the Global Fund (and other international organizations) on people *currently *on ARV. This indicator forms a key input to the epidemiological modelling package (known as Spectrum) that the UNAIDS Reference Group on Estimates, Modelling, and Projections[25] recommends for calculating lives saved and life-years gained by ARV. Life-years gained is the preferred metric for population survival benefit[25], because ARV extends life but does not cure infections.

Spectrum uses demographic data and estimates of country HIV prevalence, incidence, mortality and ARV coverage rates over time[25], to estimate epidemic trends in the presence and (hypothetical) absence of ARV in each country[24,26]. Spectrum assumes that all ARV is provided to HIV-infected people in need of such treatment, which is operationalized as a median of 3 years before estimated time of AIDS-related deaths[26]. Survival at 12 months on ARV is assumed to be 85% for both adults and children, after which each next year 5% of survivors would die[26]. People reported on treatment in 2004, 2005 or 2006 were assumed to have started in the respective years and stayed on treatment through end-2007, unless they had died. In a comparison scenario, annual mortality was assumed to be 50% by 12 months, and 100% by 24 months from starting to need ARV for people not accessing the treatment[25]. 'Lives saved' by 2007 were calculated as the difference between cumulative numbers of deaths with and without ARV.

These projections yielded average ratios of 'life-years gained per patient-year of ARV', and 'lives saved per patient on ARV', which varied among countries and over calendar years according to varying stages of disease that patients initiated ARV, indirect demographic effects (restored fertility etc.), and dynamic effects of ARV, through reduced viral load, on reducing HIV transmission. To subsequently obtain mortality effect estimates, the regionally averaged ratios of 'life-years gained per patient-year of ARV' over the period 2003-2007, and of 'lives saved per patient on ARV' at end-2007 were applied to regionally summed ARV numbers reported by Global Fund recipients at end-2007.

### TB mortality averted due to DOTS

We estimated TB mortality based on numbers of new smear-positive case detections in supported DOTS programs[18] and TB case fatality rates. For a few programs reporting case detections without specifying the smear status, we assumed that 50% of cases were smear-positive. All cases were assumed to start DOTS without delay. Because of the long duration of TB treatment, we considered half of cases detected in each calendar year to contribute to lives saved in that year, counting the other half as savings during the next year (Table 1).

For DOTS, two comparison scenarios were considered. The simplest comparison, consistent with the 'no intervention' counterfactual scenario for ARV and ITNs, was against no treatment. The alternative is against non-DOTS treatment, which is commonly used in TB impact evaluations[12,27], as DOTS programs provide better treatment to patients who would otherwise have been treated in sub-standard ways[28]. While not always clearing infection and stopping transmission, non-DOTS treatment still often prevents the patient from dying[28].

Case fatality rates for DOTS, non-DOTS and no treatment scenarios were available from WHO, stratified by HIV and smear status. These country-specific rates were combined with country-specific proportions of DOTS-treated smear-positive TB cases that are HIV-positive, for the year 2006[28].

### Malaria-related mortality averted due to ITNs

In areas of sub-Saharan Africa with stable endemic *falciparum *malaria, effective use of ITNs results in averting 5.5 under-five deaths per 1000 children protected per year[29] (Table 1). This efficacy was applied to estimate mortality reductions resulting from ITN deliveries in recipient programs in sub-Saharan Africa and in Papua New Guinea, where *falciparum *malaria occurs of a similar endemicity[30]. Mortality among adults, from non-*falciparum *malaria and in places with non-stable *falciparum *malaria was not estimated, due to limited evidence of intervention effectiveness and lack of reliable estimates of populations at risk[30].

We assumed that each ITN distributed resulted in an average of 1.1 child-year of protection, over the lifetime of the net. This assumption was based on:

• A median ratio of 0.73 between the proportion of households possessing one or more ITNs and the proportion of children under-5 that sleep under an ITN. This was the ratio observed across 37 DHS and MICS surveys conducted in malaria-endemic countries between 2000 and 2006[31];

• An effective ITN lifespan of 1.5 years. This takes into account that some Global Fund-supported malaria programs also supported ITN re-impregnations.

• 0.73 * 1.5 = 1.1.

The cumulative years of protection achieved per ITN up to the date was calculated from the dates of ITN distributions in each country: ITNs distributed before end-2006 were counted as having been used for 1.5 years, and ITNs distributed in 2007 counted for 0.5 years (see also Table 2 and Additional file 1, Online Appendix).

### Uncertainty ranges

95% uncertainty ranges (UR's) were calculated applying the delta method[32] to the calculation of averted deaths, keeping only first-order terms in the error expression and combining 95% UR's of all input parameters (see Table 1).

For service delivery results, on-site verification had revealed that in 2005-6 83% of country reports were within a 20% margin, without systematic bias in either direction (over-reporting or under-reporting)[20]. We therefore assumed a 25% error on each country delivery result, without dependency among countries.

For other parameters, errors were (conservatively) assumed to be systematic rather than country-specific. When summing lives saved across the 3 diseases, errors in disease-specific estimates were taken as independent.

## Results

### HIV/AIDS

**Figure 1:**
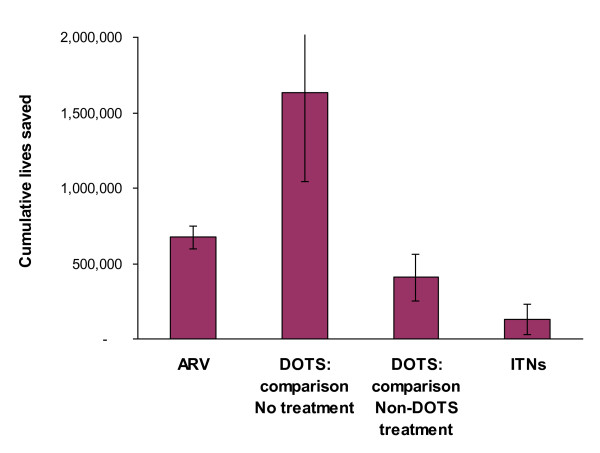
**Estimated lives saved by Global Fund-supported ARV, DOTS and ITNs, cumulatively between 2003 and end-2007**. Notes: • For DOTS, two alternative estimations are shown that differ in the counterfactual scenarios (see description in Methods). • Not included in the lives saved estimations are the - relatively small - numbers of service deliveries through multi-country grants (see Tables 3, 4 and 5). • Error bars represent 95% uncertainty ranges on the estimates, as described in Methods. ARV = antiretroviral treatment; DOTS = directly observed TB treatment, short course; ITN = insecticide-treated mosquito net.

As of end-2007, 1.44 million people were reported to be on ARV, in 91 supported HIV programs (Table 3). The resulting number of life-years gained was 1,097,000 (95% uncertainty range 993,000-1,249,000). With the 1.44 million patients alive, 108,000 were estimated to have died by end-2007, whereas, had ARV not been provided, 782,000 would have died. An estimated 681,000 deaths (619,000-774,000) were averted, or 681,000 lives saved, due to ARV scale-up by end-2007 (Figure 1).

**Table 3:** Estimation of lives saved by Global Fund-supported ARV cumulatively between 2003 and December 2007.

Region	Countries withGF-supportedARV programme	People currently on ARV, end-2007	Cumulative life-years on ARV,2003-2007	Average lives saved per person currently on ARV, end-2007*	
East Africa	11	460,228	807,514	0.466	
Western Africa	17	127,344	257,022	0.466	
Southern Africa	9	496,449	864,069	0.466	
North Africa & Middle East	10	21,383	28,104	0.466	
South Asia	4	102,545	123,989	0.502	
East Asia and The Pacific	8	157,602	349,257	0.502	
Eastern Europe & Central Asia	16	17,667	28,165	0.352	
Latin America & Caribbean	16	64,401	138,682	0.447	

**Global total**	**91**	**1,447,619**	**2,596,804**	**0.466**	
95% Uncertainty range		(1,410,000-1,486,000)			

					
					

**Region**	**Lives saved by end-2007**		Average life-years gained per patient-year of ARV, 2003-7*	**Life-years gained up to end-2007**	
		**(95% UR)**			**(95% UR)**

East Africa	214,538	(201,129-241,355)	0.424	342,386	(308,860-392,364)
Western Africa	59,362	(55,652-66,782)	0.424	108,977	(99,488-124,118)
Southern Africa	231,423	(216,959-260,351)	0.424	366,365	(328,202-421,405)
North Africa & Middle East	9,968	(9,345-11,214)	0.424	11,916	(10,715-13,679)
South Asia	51,505	(37,599-67,987)	0.443	54,927	(38,585-73,797)
East Asia and The Pacific	79,159	(57,786-104,489)	0.443	154,721	(110,765-206,085)
Eastern Europe & Central Asia	6,224	(4,419-9,634)	0.314	8,844	(6,220-13,722)
Latin America & Caribbean	28,813	(21,898-44,314)	0.351	48,677	(36,605-75,042)

**Global total**	**681,000**	(619,000-774,000)	**0.409**	**1,097,000**	(993,000-1,249,000)

ARV service delivery results are from country reports to Global Fund secretariat. The table excludes multi-country grants in the Americas and Western Pacific. Country groupings, including certain regions, are as per the Global Fund regional categorizations www.theglobalfund.org. HIV survival assumptions from the UNAIDS 2007 consensus epidemiological model package [24]. Proportional errors are shown for the two sources of error considered: mortality assumptions used in the Spectrum demographic model [26].

* weighted by country population size.

UR = 95% Uncertainty range.

### Tuberculosis

Supported TB programs in 68 countries reported a cumulative 3.3 million smear-positive TB case detections by end-2007 (Table 4). Out of these, 272,000 were estimated to have died in spite of DOTS. Using 'without-treatment' as comparison scenario, deaths would have amounted to 1.9 million, resulting in an estimated 1.63 million lives saved (1.09 - 2.17 million). Under non-DOTS treatment, by contrast, 680,000 smear-positive cases would have died, corresponding to an estimated 408,000 lives saved (265,000-551,000) (Figure 1).

**Table 4:** Estimation of lives saved by DOTS case detections and treatments in Global Fund-supported programs, cumulatively between 2003 and December 2007.

Region	Countries with GF-supported DOTS programme	Case detections in GF-supported DOTS programmes	Estimate d treatments completed	Cumulative lives saved (95% UR)			
				***Counterfactual scenario:***		***Counterfactual scenario:***	
				***No treatment***		***Non-DOTS treatment***	
East Africa	7	448,243	363,794	223,574	(128,704-318,445)	76,019	(42,182-109,856)
Western Africa	10	109,806	87,668	53,692	(31,794-75,591)	18,205	(10,396-26,015)
Southern Africa	6	239,144	208,153	133,546	(75,230-191,862)	46,957	(25,593-68,320)
North Africa & Middle East	9	68,195	45,438	26,634	(15,708-37,560)	6,342	(2,912-9,772)
Eastern Europe & Central Asia	10	72,345	53,023	26,125	(15,558-36,691)	1,530	(370-2,690)
South Asia	7	554,726	382,333	230,612	(133,671-327,553)	75,654	(41,271-110,037)
East Asia and the Pacific	9	1,774,876	1,524,979	916,232	(541,056-1,291,408)	180,256	(94,065-266,448)
Latin America & Caribbean	10	44,926	35,418	21,205	(12,588-29,823)	3,182	(1,620-4,743)

**Total**	**68**	**3,312,261**	**2,700,805**	**1,632,000**		**408,000**	
**95% Uncertainty range**		(3,211,843 -3,412,679)	(2,618,925 -2,782,685)	(1,094,000-2,169,000)		(265,000-551,000)	

DOTS results are from country reports to Global Fund secretariat. Table excludes a multi-country grant in the Western Pacific. Country groupings, including certain regions, are as per the Global Fund regional categorizations www.theglobalfund.org. Country-specific assumptions on proportions of smear-positive TB cases that are HIV-infected, and case fatality rates with and without DOTS were provided by the WHO Stop TB department (unpublished data).

### Malaria

By December 2007, 46 million ITNs had been distributed across 62 countries. Over 38 million ITNs were distributed in 35 African countries with stable endemic *falciparum *malaria; 231,000 in Papua New Guinea, and 7.4 million in lower-endemic areas (Table 5).

**Table 5:** Estimation of child-years of protection achieved with Global Fund-supported ITN deliveries and associated lives saved from malaria cumulatively between 2003 and December 2007.

Region	Countries	ITNs distributed	Cumulative child-years of protection, by end-2007	Cumulative lives saved	
					(95% UR)
**East Africa**		**22,520,505**	**13,603,974**	**66,806**	(11,134-122,477)
	Burundi	1,055,968	787,845		
	Comoros	93,000	46,163		
	CONGO, Dem. Rep.	1,040,995	786,925		
	Eritrea	119,522	120,821		
	Ethiopia	9,449,834	6,418,971		
	Kenya	3,354,177	446,860		
	Madagascar	2,639,436	2,770,712		
	Rwanda	2,471,837	648,463		
	Tanzania incl. Zanzibar	2,295,736	1,577,214		
**Western Africa**		**7,084,987**	**6,217,647**	**28,896**	(5,293-52,499)
	Benin	359,371	400,141		
	Burkina Faso	395,798	411,034		
	Cameroon	1,159,084	797,029		
	Gabon	196,611	143,482		
	Gambia	194,861	148,786		
	Ghana	2,012,569	2,150,717		
	Guinea	65,500	73,994		
	Guinea-Bissau	66,471	61,153		
	Liberia	491,225	237,882		
	Nigeria	1,099,384	863,552		
	Sao Tome & Principe	53,974	9,771		
	Senegal	445,470	351,348		
	Sierra Leone	124,669	107,617		
	Togo	420,000	461,141		
**Southern Africa**		**5,218,836**	**2,627,030**	**15,484**	(2,408-28,560)
	Angola	2,999,000	477,524		
	Malawi	2,390,000	247,799		
	Mozambique	3,620,000	345,290		
	Namibia	249,000	124,970		
	Swaziland	147,000	33,111		
	Zambia	1,993,000	1,185,976		
	Zimbabwe	1,706,000	212,361		
					
**North Africa & Middle East ($$)**		**3,480,253**	**3,382,288**	**17,167**	(2,372-31,961)
	Mali	219,985	240,464		
	Mauritania	158,000	138,631		
	Niger	2,120,092	2,207,591		
	Somalia	200,668	226,690		
	Sudan	486,433	387,283		
	Yemen	295,075	*Not included in Lives saved estimation*		
**East Asia (#)**		**4,505,913**		**1,391**	(117-2,665)
Papua New Guinea		231,000	260,955		
**South Asia**		**2,178,314**	*Not included in Lives saved estimation*		
**Eastern Europe & Central Asia**		**48,400**	*Not included in Lives saved estimation*		
**Latin America & Caribbean**		**602,895**	*Not included in Lives saved estimation*		

**TOTAL**		**45,233,035**			
**TOTAL: sub-Saharan Africa + PNG**		**38,240,506**	**25,910,265**	**130,000**	(27,000-232,000)

ITN results are from country reports to Global Fund secretariat. Table excludes multi-country grants in the Western Pacific and Andean Americas. Country groupings, including certain regions, are as per the Global Fund regional categorizations www.theglobalfund.org. PNG = Papua New Guinea.

^$$^In the North Africa & Middle East region, Yemen was included in the regional totals for ITN numbers and for population at risk, but not in the estimation of Lives saved. Yemen was not included in the total for 'sub-Saharan Africa + PNG'.

^# ^All 9 East Asia countries were included in ITN numbers, but only Papua New Guinea was included in the estimation of Lives saved.

* Weighted by country population size.

Example calculations of child-years of protection achieved and lives saved by country-reported ITN distributions are given in Additional file 1.

Across sub-Saharan Africa and Papua New Guinea, the average duration of past usage of ITNs distributed was 0.89 years by end-2007, resulting in a cumulative total of nearly 26 million of child-years of protection by end-2007 (Table 5).

These 36 countries had around 3.8 million under-5 deaths in 2006. Against this baseline, we estimated that Global Fund-supported ITNs had averted 130,000 deaths (27,000-232,000) up to end-2007 (Figure 1).

### Total lives saved

Adding the estimated lives saved from ARV, DOTS and ITN, without adjustment for interactions between diseases or the possibility of persons benefiting from multiple interventions, total lives saved by Global Fund-supported programs would stand at around 2.44 million (1.74-3.18 million) by December 2007. Alternatively, when using non-DOTS treatment as comparison (instead of no treatment), lives saved sum to 1.22 million (911,000-1,557,000).

## Discussion

The presented estimates of lives saved and life-years gained by Global Fund-supported ARV, DOTS and ITNs indicate the magnitude of mortality reductions achieved by recipient programs. While not being an actual measurement or formal evaluation of program impact, the estimations allow the Global Fund to gauge progress as service delivery reports from programs accumulate.

The estimates do not reflect the whole spectrum of interventions supported. Notably, HIV infections averted by prevention services will in future save lives from AIDS, but cannot readily be estimated from routine delivery reports. Effective HIV prevention often results from a multi-sectoral package of services[33-36], and infections prevented for a given level of intervention coverage vary among countries depending on local HIV incidence rates and transmission modes. Methods to estimate HIV infections averted need urgent development[37].

For malaria, artemisinin-based combination treatments (ACTs) probably have important mortality effects. By December 2007, Global Fund-supported programs had delivered over 24 million ACT courses, across 49 countries. The effectiveness of ACTs against mortality has not been established through randomized trials and will depend on the timeliness of and compliance with treatment; proportions of ACTs for true malaria episodes (rather than misused for non-malarial fevers); possible effects of ACTs on preventing repeat episodes due to recrudescent infection; and the comparison scenario (no treatment or ineffective mono-therapy). The presented model furthermore excluded indoor residual spraying (IRS). As more knowledge becomes available, ACT and IRS should be included in lives saved estimation.

The gap-filling nature of Global Fund financing makes it difficult to define a specific contribution as distinct from other donors and (pre-)existing in-country efforts. Some programs, particularly HIV and TB, report program-wide results covering all contributions from multiple domestic and international funding sources to the Global Fund, which promotes program supports rather than a project approach. Lives saved result from collective efforts of in-country programs, to which the Global Fund contributes alongside other partners. For malaria, on the other hand, total service deliveries covering all partners' contributions considerably outnumber results reported to the Global Fund: e.g. in 2006 alone, 63 million ITNs were produced worldwide[31]. For ARV, the 1,439,000 life-years saved represents around 33% of a total 3.2 million estimated life-years saved in low- and middle-income countries by end-2007[24]. The 33% share is somewhat less than approximately 50% share in ARV results (a total of 2.9 million as of end-2007[24]), because some large ARV programs with support from Global Fund (e.g., Brazil) had provided people on ARV much longer, with larger effects.

Several limitations and caveats concerning input data and assumptions must be acknowledged. Errors may occur at any stages. Misclassification of smear positivity for TB is one example. While service delivery numbers may be imprecise and performance-based funding may provide incentives for over-reporting, the errors are not systematically in one direction, so that aggregated portfolio results should roughly reflect what was delivered[20]. In reality, results at mid- and end-year released by the Global Fund secretariat are significantly conservative because results always have reporting delays but only actual results available at the time of data compilation are aggregated without projecting to release dates. Aggregation at country level is done also in a conservative manner to avoid double counting. Data quality issues nevertheless highlight an urgent need to strengthen national monitoring and health information systems.

The presented methodology was designed exclusively to produce a portfolio-aggregate indication of global-level health gain, although we used certain country-specific input assumptions and presented estimates by regional break-down. Country-specific evaluations would, in contrast, require more refined methods and inputs.

The largest uncertainties in these estimates lie in the epidemiological models and their basic (cross-country) assumptions. The 2007 UNAIDS model for estimating HIV-related deaths[25] may be possibly optimistic in assuming 5-15% annual mortality on ARV, since cohort studies in resource-poor settings found up to 27% mortality within the first year, and substantial losses to follow-up which may include some additional mortality[38,39]. Given that ARV reporting to Global Fund does not include an explicit dimension of treatment quality or patient retention, some proportion of treatments delivered are likely to be of less good quality and clinical outcome than is typical in western settings or in research cohorts in resource-poor settings.

For TB, lives saved depend critically on the comparison scenario (Figure 1). While a simplistic comparison against 'no-treatment' is most consistent across the services studied, this scenario may be less appropriate for DOTS (compared to ARV and ITNs), given the relatively high effectiveness and potential availability of non-DOTS treatment[12,27,28]. The purpose of the paper is to indicate the overall scale of programs rather than additional impact due to scale-ups.

Our estimate of lives saved by Global Fund-supported ITNs depends critically on assumptions to convert ITN deliveries into child-years of protection. The assumption that each ITN distributed provides an average 1.1 child-year of protection (see Methods) is simplistic and probably conservative, because: (i) some 38% of ITNs distributions reported by supported programs were long-lasting insecticidal nets (LLINs) that remain effective throughout their lifetime of typically 3-4 years[40]; (ii) even for conventional ITNs, the assumed 1.5-year lifespan may be short in view of insecticide re-impregnations supported through Global Fund grants[41], and (iii) ITN usage was estimated based on 2000-06 surveys, but increased funds also support behavior change communication activities. Estimates of child protection by ITNs should be validated and improved based on country-specific coverage data, forthcoming from household surveys in many recipient countries. Since recent surveys demonstrated significant increases in ITN usage among under-fives[31,42], lives saved from malaria are expected to increase rapidly over the coming years.

There is reasonable consensus about the efficacy of ITNs against mortality in children exposed to endemic *falciparum *malaria, for given levels of coverage, in all recent malaria impact models[14], including a consensus model under development by UNICEF and WHO[43]. Malaria effect estimates were, nonetheless, conservative because we did not consider mortality averted outside sub-Saharan Africa and Papua New Guinea. In any case, considerable uncertainties about the size and geographical distribution of populations exposed to stable endemic malaria[30] contribute to relatively wide uncertainty ranges, compared to ARV and DOTS (error bars in Figure 1).

The presented uncertainty ranges, particularly for ARV and DOTS, may be minimum intervals, which do not capture the complex chain of factors that intervene between service delivery counts, population coverage and impact, such as quality of services including (mis-)diagnosis, drug quality, patient counselling and support to promote patient retention and treatment adherence. Periodic evaluation of quality of services must therefore be added to program progress tracking and performance evaluation.

Longer-term, improved approaches to measuring disease impact are essential in low- and middle-income countries. To enable this and to evaluate its institutional performance and partners context, the Global Fund recommends that programs spend 5-10% of grant budgets on strengthening monitoring and evaluation systems as well as supporting vital registration. Furthermore, the Global Fund Board has commissioned an independent Five-Year Evaluation including impact evaluations reinforcing quality data collection and analysis in 20 countries[20,44].

## Conclusion

The presented estimates indicate the scale of mortality effects that Global Fund-supported programs may have achieved over recent years. While the numbers carry great margins of uncertainty and cover only selected program components, they reflect the best information available to date. Evidence-based evaluation of the impact of HIV/AIDS, TB and malaria programs supported by the Global Fund and other partners is ongoing and must be further strengthened to maximize health gain. Great improvements will be made by triangulating service delivery reports with population-level data on intervention coverage and demographic outcomes, information on quality of services, and trends in health facility disease burdens recorded in national health information and management systems.

## Competing interests

No particular funding was provided to develop this manuscript. The authors declare that they have no competing interests.

## Authors' contributions

RK, EK and DLB designed the study, led the analyses, and developed the manuscript. CD and CW oversaw the TB estimation and contributed to interpretation and writing. EK implemented the malaria estimation, with advise from RS and BN who also contributed to interpretation and writing. RL and JMGC contributed to estimating and interpreting the effects of ARV and to writing. JC and BS conceptualized and contributed to interpretation and writing. All authors read and approved the final manuscript.

## Pre-publication history

The pre-publication history for this paper can be accessed here:

http://www.biomedcentral.com/1471-2334/10/109/prepub

## Supplementary Material

Additional file 1 **Example calculation of lives saved by ITNs**. (a) basic calculation; (b) additional step to correct for 'over 100% child coverage' in selected countries. Click here for file
