# Supplementary material for: Lives saved by Global Fund-supported HIV/AIDS, tuberculosis and malaria programs: estimation approach and results between 2003 and end-2007
Source: BMC Infect Dis. 2010 Apr 30;10:109. doi: 10.1186/1471-2334-10-109 (PMC2876166; doi:10.1186/1471-2334-10-109)
Supplement: Additional file 1 — Example calculation of lives saved by ITNs. (a) basic calculation; (b) additional step to correct for 'over 100% child coverage' in selected countries. [file 1471-2334-10-109-S1.DOC]

**Online Appendix with manuscript
‘***Lives saved by Global Fund-supported … services…*’, by Komatsu-R et al..

**Example calculation of lives saved by ITNs.
(a) basic calculation for Burundi;
(b) additional step to correct for ‘over 100% child coverage’ in selected countries.**

Table (a) illustrates the calculation of lives saved by GF-supported ITN distributions, for the example of Burundi. The calculation method is described in detail in the Methods section of the main text and in Table 1.

**Table (a).** Burundi: basic calculation

|  |  | *2005* | *2006* | *2007* | *Total* |
| --- | --- | --- | --- | --- | --- |
| ITNs distributed | In specified year | 264,991 | 322,783 | 468,194 |  |
|  | Cumulative by end of specified year | 264,991 | 587,774 | 1,055,968 |  |
| Average duration of use per ITN distributed, by end-2007 | | 1.5 year | 1.5 year | 0.5 year |  |
| Lives saved by end-2007 | | 264,991 * 1.5 * 5.5/1000 * 73% | 322,783 * 1.5 * 5.5/1000 * 73% | 468,194 * 0.5 * 5.5/1000 * 73% |  |
|  | | = 1,596 | = 1,944 | = 940 | **4,480** |

An additional calculation step was needed for Rwanda and Sao Tome & Principe, where − unlike for all other countries − the cumulative numbers of ITNs distributed by end-2007 exceeded the estimated need to cover all children under-5. In table (b), this is evident from an over 100% ‘proportion of child-nights protected’ (a cross-sectional coverage measure, as distinct from cumulative child-years of protection conferred by ITNs over a specified duration).

To arrive at balanced estimates of lives saved for these two countries, the respective initial lives saved estimates (calculated as shown in Table ‘a’ for Burundi) were adjusted downward by dividing them by their respective 216% and 202% ‘uncorrected proportions of child-nights protected’.

**Table (b).** Rwanda and Sao Tome & Principe: additional calculation step

|  |  | *Burundi* | *Rwanda* | *Sao Tome & Principe* |
| --- | --- | --- | --- | --- |
| Population size (2007) | All, all-age | 8,508,229 | 9,724,575 | 157,637 |
|  | At malaria risk*, all-age | 5,660,000 | 5,030,000 | 130,000 |
|  | At malaria risk*, under-5 | 994,250 | 834,912 | 19,542 |
| ITNs distributed, cumulatively by end-2007 | | 1,055,968 | 2,471,837 | 53,974 |
| Proportion of child-nights protected | Uncorrected | 1,055,968 / 994,250 * 73% of ITNs used for child under-5 =  **78%** | 2,471,737 / 834,912 * 73% of ITNs used for child under-5 = **216%** | 53,974 / 19,542 * 73% of ITNs used for child under-5 = **202%** |
|  | Corrected for maximum 100% coverage | **78%** | **100%** | **100%** |
| Lives saved | Uncorrected | 4,480 | 7,708 | 108 |
|  | Corrected for maximum 100% coverage | **4,480** | **3,567** | **54** |

* Proportions of country populations exposed to stable endemic *falciparum* malaria were derived from ^1^. We assumed same proportions at risk for populations of all ages and for under-fives.

**Reference for Online Appendix**

1. Guerra CA, Gikandi PW, Tatem AJ, et al. The limits and intensity of Plasmodium falciparum transmission: implications for malaria control and elimination worldwide*. PLoS M*ed 200**8**;5(**2**):e38.
